# Influence of Nutrient Intake and Oral Health Among Adolescents in Mallorca: A Cross-Sectional Study

**DOI:** 10.3390/children13101311

**Published:** 2026-09-26

**Authors:** Pablo Estebala Alández, Irene Coll Campayo, Daniela Vallejos-Rojas, María Luisa Bonet, Nora López-Safont

**Affiliations:** 1 Faculty of Dentistry, ADEMA University School, C. Passamaners 11, 07009 Palma, Spain; p.estebala@eua.edu.es (P.E.A.); i.coll@eua.edu.es (I.C.C.); d.vallejos@eua.edu.es (D.V.-R.); 2 Adema-Health Group of University Institute for Research in Health Sciences (IUNICS), 07211 Palma, Spain; 3 Nutrigenomics, Biomarkers and Risk Evaluation (NuBE) Research Group, University of the Balearic Islands, 07122 Palma, Spain; luisabonet@uib.es; 4 CIBER Fisiopatología de la Obesidad y Nutrición (CIBEROBN), Instituto de Salud Carlos III (ISCIII), 28029 Madrid, Spain; 5 Artificial Intelligence Research Institute of the Balearic Islands (IAIB), 07122 Palma, Spain; 6 Institut d’Investigació Sanitària Illes Balears (IdISBa), 07120 Palma, Spain

**Keywords:** adolescents, periodontal disease, diet quality, micronutrients, fiber, antioxidants, vitamin C, selenium, oral health, public health

## Abstract

**Highlights:**

**What are the main findings?**

- Periodontally healthy adolescents showed significantly higher intakes of dietary fiber, magnesium, selenium, vitamin A, and vitamin C than adolescents with periodontal disease.
- Although the overall multivariable model was significant, no individual nutrient remained independently associated with periodontal disease after adjustment.

**What are the implications of the main findings?**

- The findings suggest that a more favorable overall nutritional profile, rather than the intake of a single nutrient, may be associated with better periodontal health during adolescence.
- Promoting a balanced, nutrient-dense diet during adolescence may represent a complementary strategy for supporting periodontal and overall oral health.

**Abstract:**

Background: Adolescence is a critical developmental period characterized by increased nutritional requirements and the establishment of dietary habits that may influence long-term health outcomes, including oral health. Dietary patterns with low nutrient density may contribute to inflammatory processes involved in periodontal disease. This study aimed to analyze the association between dietary intake and periodontal health status in a sample of adolescents from Mallorca, Spain. Methods: A cross-sectional epidemiological study was conducted among 156 adolescents, aged 15 years, attending secondary schools in Mallorca. Periodontal status was assessed using the Community Periodontal Index (CPI) according to the World Health Organization (WHO) Pathfinder methodology. Dietary nutrient intake was evaluated using a single 24 h dietary recall conducted following the European Food Safety Authority (EFSA) recommendations. Participants were classified as periodontally healthy or as having periodontal disease according to the presence of affected sextants. A binary logistic regression analysis was subsequently performed to evaluate the independent association between nutrient intake and periodontal disease. Results: Of the 156 adolescents included (81 girls and 75 boys), 46 were classified as periodontally healthy and 110 as having periodontal disease. No significant association was observed between sex and periodontal status (*p* = 0.695). Adolescents with healthy periodontal status had significantly higher intakes of dietary fiber (*p* = 0.011), magnesium (*p* = 0.018), selenium (*p* = 0.035), vitamin A (*p* = 0.018), and vitamin C (*p* = 0.011). Conclusions: Higher intakes of micronutrients with antioxidant and anti-inflammatory properties appear to be associated with better periodontal health among adolescents. These findings suggest that promoting nutrient-dense dietary patterns during adolescence may help support periodontal health.

## 1. Introduction

Adolescence is a developmental stage characterized by rapid physiological growth and profound physical, psychological, and social changes that mark the transition from childhood to adulthood [1,2].

Adolescents have increased nutritional requirements due to the physiological changes that occur during this period. However, dietary quality often deteriorates during adolescence, with poor dietary patterns and inadequate nutrient intakes being commonly reported [3,4]. These changes are influenced by individual, social, and environmental factors associated with increasing independence and greater interaction with the social environment [2].

Adolescence may also represent a critical period for periodontal health [5]. Epidemiological and immunological evidence suggests that the irreversible tissue damage associated with periodontal disease begins during late adolescence and early adulthood [6]. Oral conditions affecting adolescents include dental plaque-induced gingivitis, non-dental-plaque-induced gingival diseases, periodontitis (including chronic and aggressive forms), necrotizing periodontal diseases, periodontitis as a manifestation of systemic disease, periodontal abscesses, endodontic–periodontal lesions, mucogingival deformities and conditions, occlusal trauma, and peri-implant diseases [7]. Gingival disease becomes highly prevalent during adolescence, with plaque-induced gingivitis affecting almost all individuals during puberty [8]. European epidemiological studies corroborate that periodontal alterations are common during adolescence. Among Polish 15-year-olds, gingival bleeding and shallow periodontal pockets were observed in 37.4% and 2.8% of participants, respectively [9]. Similarly, among Greek 15-year-olds, 21.6% presented bleeding on probing, 44.3% dental calculus, and 3.2% periodontal pockets ≥ 4 mm [10].

In recent decades, the dietary habits of Spanish adolescents have undergone substantial changes, particularly the progressive abandonment of balanced dietary patterns [11]. Many adolescents frequently consume large amounts of refined carbohydrates, sugar-sweetened beverages, energy drinks, and ultra-processed foods, resulting in dietary patterns with low nutritional density that may contribute to the development of periodontal diseases [5,11,12,13,14]. Such dietary patterns may promote gingival inflammation, leading to clinical signs such as gingival redness and bleeding, which are characteristic of dental plaque-induced gingivitis [15].

Although the relationship between nutrition and periodontal disease has been widely investigated in adults, evidence in adolescent populations remains limited, particularly concerning individual nutrient intake. Given the physiological changes in adolescence, the high prevalence of oral diseases at this stage, and the close relationship between diet and oral health, identifying nutritional factors associated with periodontal status is especially important. Early preventive action during adolescence may help limit the persistence and progression of periodontal inflammation, thereby reducing the risk of irreversible periodontal tissue damage and tooth loss in adulthood [5,6].

In a previous study, we examined the association between processed-food consumption frequency and periodontal status [14], as consumption frequency is a relevant dimension of dietary exposure in relation to periodontal health. Building on this previous work, the current investigation provides a complementary analysis in the same source population, focused on nutrient intake and adequacy, assessed using a 24 h dietary recall. Accordingly, we aimed to compare nutrient intake between adolescents from Mallorca, Spain, with and without periodontal disease. Findings may improve the understanding of the relationship between nutritional adequacy and periodontal health during adolescence.

## 2. Materials and Methods

### 2.1. Study Design and Study Population

A cross-sectional epidemiological study was conducted, following the World Health Organization (WHO) Pathfinder methodology for oral health surveys [16], among adolescents enrolled in the fourth year of compulsory secondary education in Mallorca.

Participants were eligible if they were 15 years of age, attended one of the schools selected for the study, and had written informed consent provided by a parent or legal guardian. Exclusion criteria included age outside the established range, a severe systemic disease, and unavailability or lack of cooperation during data collection. For inclusion in the present nutritional analysis, participants were additionally required to have complete periodontal assessment data and a valid 24 h dietary recall. Consequently, the final analytical sample comprised 156 adolescents (81 girls and 75 boys).

The sampling strategy was stratified by geographical setting (urban, peri-urban, and rural areas) and school type (public and private/subsidized schools). The sampling frame was obtained from the Directorate General for Planning, Organization and Schools of the Autonomous Community of the Balearic Islands and the National Institute of Statistics. Schools were first stratified according to geographical setting (urban, peri-urban, and rural areas) and school type (public and private/subsidized schools). Within each stratum, schools were selected using systematic random sampling, applying proportional allocation to ensure that the final sample was representative of the distribution of schools in the study area. Participants were then recruited from the selected schools among 15-year-old students attending the corresponding school grade. All eligible students were invited to participate.

The study protocol was approved by the Research Ethics Committee of the Balearic Islands (CEI-IB) on 17 September 2018 (Approval No. IB3737) and was conducted in accordance with the ethical principles of the Declaration of Helsinki. All collected data were anonymized to ensure participant confidentiality. Before the study commenced, parents or legal guardians received written information about the study and signed an informed consent form. Only adolescents whose parents or legal guardians provided written informed consent were included.

### 2.2. Data Collection and Study Variables

Data on oral health, age, sex, and dietary intake were collected between November 2018 and December 2019.

Oral examinations were performed according to the WHO recommendations described in Oral Health Surveys: Basic Methods [16]. Seven calibrated dentists performed all the examinations. Before the start of the study, all examiners underwent a standardized calibration process that included both theoretical instruction and practical clinical training. Interexaminer reliability was assessed through duplicate clinical examinations, and agreement was evaluated using Cohen’s Kappa coefficient. The mean Kappa value obtained was 0.757, indicating substantial agreement between examiners. To ensure consistency and standardization throughout the clinical assessment, a reference examiner (gold standard) participated in the calibration procedure. A detailed description of the calibration protocol was published previously [17,18].

Periodontal status was assessed using the Community Periodontal Index (CPI). The number of healthy sextants, sextants with bleeding, calculus, and periodontal pockets was recorded. Six sites were examined on each index tooth (16, 11, 26, 31, 36, and 46) using the WHO periodontal probe. Each sextant was assigned the highest CPI score observed: healthy (Code 0), bleeding on probing (Code 1), or dental calculus (Code 2). Participants were classified as periodontally healthy when all sextants were healthy and as having periodontal disease when at least one sextant presented a CPI score ≥ 1.

To assess dietary intake and identify nutritional factors associated with oral health, trained dietitians collected dietary information following the recommendations of the European Food Safety Authority (EFSA) developed within the EU Menu Project [19]. Data collection followed the methodological guidelines described in General Principles for the Collection of National Food Consumption Data in the View of a Pan-European Dietary Survey [20]. The dietary assessment protocol has been previously applied in epidemiological studies [21].

The variables included in the analysis were sex (boy/girl), Community Periodontal Index (CPI), number of sextants according to CPI code (healthy, bleeding, and calculus), periodontal status (periodontally healthy or periodontal disease), and dietary nutrient intake data.

### 2.3. Nutritional Assessment

Dietary intake was assessed using a single 24 h dietary recall. Although a single 24 h dietary recall cannot estimate an individual’s habitual dietary intake because of day-to-day variation, it is an appropriate method for estimating mean nutrient intake at the group level when standardized protocols are applied. As a quality-control measure, three trained investigators cross-checked completed dietary recalls to identify possible omissions or inconsistencies before data analysis. All dietary data were entered into the Nutrium^®^ nutritional analysis software (2026). Food composition data were obtained from two Spanish databases integrated into Nutrium^®^: the Spanish Food Composition Database (BEDCA, University of Granada) and the CESNID Food Composition Database (CESNID Foundation, Spain). Vitamin A values are expressed as retinol equivalents (RE, μg), calculated as the sum of preformed retinol and provitamin A carotenoids according to conventional conversion factors: 1 μg RE = 1 μg retinol + (β-carotene/6) + (α-carotene/12) + (β-cryptoxanthin/12). Nutrient intake was subsequently analyzed according to sex and periodontal status. Compliance with the Dietary Reference Values (DRVs) established by the European Food Safety Authority (EFSA) for each sex and age group was also evaluated.

### 2.4. Statistical Analysis

Statistical analyses were performed using IBM SPSS Statistics for Windows, Version 31.0 (IBM Corp., Armonk, NY, USA). Continuous variables are presented as mean ± standard deviation (SD) or standard error (SE), whereas categorical variables are expressed as absolute frequencies and percentages. Differences in energy intake, nutrient intake, and compliance with the EFSA DRVs between periodontally healthy participants and those with periodontal disease were assessed using independent-samples Student’s *t*-tests. Differences in categorical variables were evaluated using the chi-square test.

A binary logistic regression analysis was performed to evaluate the independent association between nutrient intake and periodontal disease. Periodontal status (periodontally healthy/periodontal disease) was included as the dependent variable, whereas sex, total energy intake (kcal), dietary fiber (g), magnesium (mg), selenium (µg), vitamin A (µg RE), and vitamin C (g) were entered simultaneously as independent variables using the enter method. Odds ratios (ORs) and 95% confidence intervals (95% CIs) were calculated. The multivariable model assessed the simultaneous contribution of the selected nutrients but was not adjusted for additional demographic, behavioral, or socioeconomic variables.

The proportion of participants meeting the EFSA DRVs for each nutrient was also calculated according to sex and periodontal status. All statistical tests were two-sided, and *p* < 0.05 was considered statistically significant.

## 3. Results

### 3.1. Sample Characteristics

A total of 156 adolescents aged 15 years were included in the study between October 2018 and November 2019, comprising 75 boys and 81 girls (Table 1). According to periodontal status, 46 participants were classified as periodontally healthy and 110 as having periodontal disease. No significant association was observed between sex and periodontal status (χ^2^ test, *p* = 0.695).

### 3.2. Maximum Community Periodontal Index Score

Table 2 presents the distribution of participants according to their highest Community Periodontal Index (CPI) score. The highest CPI score recorded was calculus (40.4%), followed by bleeding on probing (25.6%), while 29.5% of participants presented a healthy periodontium (Code 0). No participants presented periodontal pockets (CPI Code 3).

### 3.3. Dietary Intake

Dietary intake differed significantly between boys and girls for energy intake, protein, carbohydrate, cholesterol, calcium, phosphorus, iron, thiamine, riboflavin, and niacin (Table 3).

### 3.4. Dietary Intake According to Periodontal Status

In univariate analyses, no significant differences were observed between periodontally healthy adolescents and those with periodontal disease in mean energy intake. Likewise, no statistically significant differences were found for protein, carbohydrate, total fat, saturated fatty acids, monounsaturated fatty acids, polyunsaturated fatty acids, or cholesterol intake (all *p* > 0.05). However, significant differences were observed for dietary fiber (*p* = 0.011), magnesium (*p* = 0.018), selenium (*p* = 0.035), vitamin A (*p* = 0.018), and vitamin C (*p* = 0.011) (Table 4).

### 3.5. Compliance with EFSA Dietary Reference Values

Mean nutrient intake and the corresponding percentage of the EFSA DRVs (Table 5) for the nutrients significantly associated with periodontal status are presented according to sex and periodontal status in Table 6.

To evaluate the adequacy of nutrient intake, the proportion of adolescents meeting the EFSA DRVs (Table 5) was calculated for the nutrients that were significantly associated with periodontal status in the univariate analysis (Table 7). Overall, compliance with the recommendations was low. However, periodontally healthy adolescents showed higher compliance with the DRVs for magnesium (26.1% vs. 11.8%), vitamin A (37.0% vs. 14.5%), and vitamin C (47.8% vs. 28.2%) than adolescents with periodontal disease. Compliance with the recommendations for dietary fiber and selenium was low in both groups.

Differences between observed nutrient intake and the EFSA DRVs according to periodontal status are presented in Table 8. Adolescents with periodontal disease showed significantly greater deficits in dietary fiber (*p* = 0.011), magnesium (*p* = 0.015), selenium (*p* = 0.035), vitamin A (*p* = 0.002), and vitamin C (*p* = 0.011) than periodontally healthy adolescents. Among the nutrients analyzed, the largest difference between groups was observed for vitamin A. Periodontally healthy adolescents met the EFSA recommendation for vitamin C on average, whereas adolescents with periodontal disease remained below the recommended intake.

### 3.6. Multivariable Analysis

A binary logistic regression analysis was performed to evaluate whether sex, total energy intake, dietary fiber, magnesium, selenium, vitamin A, and vitamin C were independently associated with periodontal disease. The results are presented in Table 9. The model was statistically significant (Omnibus χ^2^ = 15.77, *p* = 0.027), and the Nagelkerke pseudo-R^2^ value of 0.137 indicated modest explanatory capacity, accounting for approximately 13.7% of the variability in periodontal status. None of the individual variables remained independently associated with periodontal disease after multivariable adjustment (all *p* > 0.05). Selenium (*p* = 0.056) and vitamin C (*p* = 0.068) showed borderline statistical significance.

## 4. Discussion

In the present work, both study groups, periodontally healthy adolescents and adolescents with periodontal disease, showed mean deficits in dietary fiber, magnesium, selenium, and vitamin A relative to the EFSA DRVs. However, these deficits were consistently greater among adolescents with periodontal disease. Additionally, mean vitamin C intake exceeded the DRV in the periodontally healthy group but remained below the recommendation in the periodontal disease group. These differences were consistent across analyses of absolute nutrient intake, compliance with the EFSA DRVs, and nutrient deficits relative to the recommendations. Together, these findings highlight the potential importance of overall diet quality in maintaining periodontal health during adolescence.

Despite considerable advances in the prevention and understanding of periodontal diseases, they remain a major public health concern, particularly among vulnerable populations such as children and adolescents [28]. Diet plays a fundamental role in oral health, and growing evidence indicates that inadequate nutritional status is associated with an increased risk of oral diseases, including periodontal disease [29,30,31,32,33].

In this sample of 156 adolescents aged 15 years, significant differences were observed in the intake of dietary fiber, magnesium, selenium, vitamin A, and vitamin C between periodontally healthy adolescents and those with periodontal disease, with consistently higher intakes in the healthy group. These between-group differences were identified in the univariate analyses; however, none of the nutrients showed a statistically significant independent association with periodontal status in the multivariable logistic regression model. As such, these findings should be interpreted cautiously; however, they may still be relevant given that adolescence is a critical period characterized by rapid somatic growth, pubertal maturation, and neurocognitive development, processes that are accompanied by increased nutritional requirements and may increase vulnerability to unhealthy dietary patterns [2,14]. Notably, total energy intake did not differ significantly between groups (1645.8 ± 452.6 vs. 1559.2 ± 524.6 kcal/day; *p* = 0.330). Therefore, the higher intake of these nutrients among periodontally healthy adolescents likely reflects greater dietary nutrient density rather than higher overall energy intake, suggesting that diet quality may be more relevant for periodontal health than the quantity of food consumed.

Although significant differences in dietary intake were observed between boys and girls, no significant differences were found in the prevalence of periodontal disease by sex (72.0% in boys vs. 69.1% in girls; *p* = 0.695). Similarly, sex was not independently associated with periodontal disease in the multivariable logistic regression model. The higher energy intake observed among boys (1683.9 ± 539.5 kcal/day) compared with girls (1492.8 ± 454.2 kcal/day) is consistent with the higher energy requirements of male adolescents during growth. This physiological difference may explain why the observed sex differences in nutrient intake did not translate into differences in periodontal health status.

Nutrient intakes below the EFSA DRVs may contribute to impaired growth, development, and compromised immune function during adolescence [34]. This compromised immune function may be particularly relevant to periodontal disease, a chronic inflammatory condition driven by the interaction between the oral biofilm and the host immune response. Current evidence indicates that adequate nutrition plays an important role in modulating inflammatory responses, supporting immune function, promoting tissue repair, and maintaining antioxidant defenses, all of which contribute to periodontal homeostasis. Consequently, insufficient intake of essential nutrients may disrupt these biological processes, increasing susceptibility to periodontal inflammation [35].

Nevertheless, despite growing interest in the relationship between nutrition and periodontal health, studies conducted specifically in adolescents remain scarce. Consequently, evidence regarding the role of dietary intake in the early stages of periodontal disease remains limited. In this context, our findings provide further evidence that adolescents with periodontal disease not only had lower absolute intakes of dietary fiber, magnesium, selenium, vitamin A, and vitamin C but were also less likely to meet the EFSA DRVs for these nutrients. This finding reinforces the hypothesis that the overall nutritional adequacy and quality of the diet, rather than the intake of individual nutrients, may be associated with periodontal health during adolescence.

The higher dietary fiber intake observed among periodontally healthy adolescents is consistent with previous evidence linking greater fiber consumption during childhood and adolescence with higher overall diet quality and more favorable metabolic profiles [36,37]. Several studies have shown that adequate fiber intake during these life stages is associated with a lower risk of cardiometabolic disease, type 2 diabetes, and other non-communicable diseases later in life [36,38]. Moreover, diets low in dietary fiber are often characterized by a higher consumption of ultra-processed foods and free sugars, which have been widely associated with dental caries and oral biofilm dysbiosis [39]. In addition, fiber-rich foods require greater mastication and stimulate salivary flow, mechanisms that contribute to acid neutralization and the maintenance of oral homeostasis [6]. Previous studies have also reported that dietary fiber intake is frequently below recommended levels among adolescents [36,37], which may partially explain the higher prevalence of oral diseases observed in this age group compared with other age groups [40,41]. The present findings are consistent with this evidence and further support the importance of promoting adequate dietary fiber intake during adolescence.

Magnesium is an essential mineral involved in numerous biological processes, including immune regulation, bone metabolism, and antioxidant defense. Previous studies have shown that higher magnesium intake and a more favorable magnesium-to-calcium (Mg/Ca) ratio are associated with reduced periodontal probing depth (PPD), lower clinical attachment loss (CAL), and a lower prevalence of periodontitis. Longitudinal studies have further demonstrated that a higher Mg/Ca ratio is associated with a slower progression of clinical attachment loss and a reduced risk of tooth loss, even among individuals with elevated systemic inflammatory markers [42,43,44]. These findings suggest that magnesium may exert protective effects through its anti-inflammatory and antioxidant properties, as well as its role in maintaining bone homeostasis, which is particularly relevant given the inflammatory nature of periodontal disease [43]. In the present study, mean magnesium intake was 234.8 mg/day in periodontally healthy adolescents and 201.8 mg/day in those with periodontal disease. These values were lower than the median usual intakes reported in the ENALIA study among Spanish adolescents aged 14–17 years: 317 mg/day in boys and 254 mg/day in girls [21].

Selenium, although required only in trace amounts, is an essential micronutrient involved in the antioxidant defense system through its role in selenoproteins, including glutathione peroxidases and thioredoxin reductases [45,46]. These enzymes play a fundamental role in protecting tissues against oxidative stress and regulating inflammatory responses. Previous studies have reported that lower selenium levels are associated with an increased risk of periodontitis [45,46]. This association has been attributed to a reduced antioxidant capacity, leading to increased oxidative stress and a greater susceptibility to chronic inflammatory conditions such as periodontal disease [46]. The lower selenium intake observed among adolescents with periodontal disease in the present study is consistent with these findings and further supports a potential role of selenium in maintaining periodontal health. In the present study, mean selenium intake was 31.4 µg/day in the periodontally healthy group and 21.1 µg/day in the group with periodontal disease, whereas ENALIA reported higher median intakes of 129.8 µg/day in boys and 103.8 µg/day in girls [21]. Nevertheless, these comparisons should be interpreted cautiously because ENALIA estimated usual intake using two non-consecutive 24 h recalls, whereas the present study used a single recall.

Vitamin A plays a crucial role in maintaining the integrity of epithelial tissues and mucosal barriers and contributes to immune regulation through its immunomodulatory and antioxidant properties [47,48]. These functions are particularly relevant to periodontal tissues, where an adequate epithelial barrier and a balanced immune response are essential for maintaining periodontal health. A recent meta-analysis suggested that vitamin A supplementation may protect against periodontal disease, although the available evidence remains limited and further well-designed studies are needed to confirm this association [47]. Similarly, observational studies have reported an inverse association between vitamin A status and periodontal disease risk [49]. Consistent with these findings, adolescents with periodontal disease in the present study showed significantly lower vitamin A intake than periodontally healthy participants, reinforcing the importance of meeting the recommended dietary intake during adolescence. Notably, among the nutrients examined, vitamin A showed the greatest between-group difference in deficit relative to the DRVs, with adolescents with periodontal disease exhibiting the largest shortfall, further emphasizing its potential relevance to periodontal health.

Vitamin C is one of the main dietary antioxidants and plays an essential role in collagen synthesis, wound healing, and the maintenance of connective tissue integrity. It also acts as a cofactor for numerous enzymes involved in antioxidant defense and immune function, contributing to the protection of periodontal tissues against oxidative damage [50]. Vitamin C deficiency has been associated with gingival bleeding, impaired collagen formation, delayed wound healing, and an increased susceptibility to periodontal disease [51]. Previous studies have reported that low vitamin C intake and reduced plasma vitamin C concentrations are associated with a higher prevalence and severity of periodontal disease [50,52,53]. In the present study, vitamin C intake was higher among periodontally healthy adolescents (107.1 mg/day vs. 75 mg/day in the periodontally diseased group), modestly exceeding the EFSA DRV. This intake can easily be achieved through commonly consumed fruits and vegetables. However, the specific food sources contributing to participants’ habitual vitamin C intake cannot be determined from the single 24 h dietary recall used in this study.

The available evidence supports the important role of nutrition in maintaining periodontal health. A healthy dietary pattern that provides adequate amounts of essential nutrients is considered fundamental for preserving oral health [54]. Although several micronutrients, including vitamin A, B-complex vitamins, vitamin C, calcium, and zinc, have been proposed as particularly relevant in the prevention of periodontal disease [53,54], other studies have identified different nutrients as potential protective factors [55]. These findings highlight the complexity of the relationship between diet and periodontal health. In our study, although several nutrients were significantly associated with periodontal status in the univariate analyses, none remained independently associated after multivariable adjustment. This finding suggests that the relationship between nutrient intake and periodontal disease is more likely explained by the overall dietary pattern than by the isolated effect of a single nutrient. Because dietary fiber, antioxidant vitamins, and essential minerals are highly correlated within healthy dietary patterns, their combined effect may be more relevant than the contribution of any individual nutrient.

The present study has several strengths. It is one of the few studies evaluating the association between nutrient intake and periodontal health in adolescents using standardized WHO periodontal examinations together with dietary assessment based on EFSA recommendations. An additional strength of the present findings is that the observed associations were not limited to differences in absolute nutrient intake. When nutrient intake was evaluated relative to the age- and sex-specific EFSA DRVs, adolescents with periodontal disease presented greater deficits for dietary fiber and several micronutrients. These findings further support the hypothesis that overall diet quality may be more relevant to periodontal health than the total quantity of food consumed.

Nevertheless, several limitations should be acknowledged. First, the cross-sectional design precludes establishing temporal or causal relationships between dietary intake and periodontal status. Therefore, the findings should be interpreted as associations within the study population rather than evidence of a causal effect. Second, the small-to-moderate sample size may have limited the statistical power, particularly in the multivariable analysis, as well as the generalizability of the findings. Third, although periodontal status was assessed using the standardized WHO Community Periodontal Index (CPI), this approach may not capture the full extent and severity of periodontal disease, and the affected group may include participants with different degrees of periodontal involvement. Fourth, dietary intake was assessed using a 24 h dietary assessment, which may not fully reflect participants’ usual intake due to day-to-day variability and potential reporting bias. Finally, residual confounding cannot be excluded, as additional dietary, behavioral, socioeconomic, and oral-health-related factors may influence the observed associations. Future prospective cohort studies should include larger and more diverse adolescent populations, a comprehensive nutritional assessment at baseline, and repeated nutritional and periodontal examinations, including annual CPI assessments, to evaluate changes in periodontal status over time and clarify the temporal relationship between dietary factors and periodontal health during adolescence.

## 5. Conclusions

Findings suggest a potential association between diet quality and periodontal health among 15-year-old adolescents. Periodontally healthy participants showed higher intakes of dietary fiber, magnesium, selenium, vitamin A, and vitamin C, together with greater compliance with the EFSA DRVs, than adolescents with periodontal disease. However, these differences were observed only in the univariate analyses, and the cross-sectional design prevents establishing causal relationships. Taken together, the findings suggest that overall dietary patterns characterized by higher nutrient density, rather than the intake of individual nutrients alone, may be relevant to periodontal health during adolescence. Further research is needed to investigate the potential role of a balanced, nutrient-dense diet in promoting oral health and preventing periodontal disease among young populations. Because adolescence is a critical period for establishing lifelong dietary habits, nutritional assessment at this stage and nutrition education may represent complementary components of oral health promotion and periodontal disease prevention.

## Figures and Tables

**Table 1:** Sample distribution by sex and disease status.

		Healthy (*n*)	Affected (*n*)	Total (*n*)	*p*-Value
Sex	Boys	21	54	75	0.695
	Girls	25	56	81	
Total		46	110	156	

(*n*, sample size).

**Table 2:** Distribution of participants according to the highest Community Periodontal Index (CPI) score.

Maximum CPI	*n*	%
0 (Healthy)	46	29.5%
1 (Bleeding)	40	25.6%
2 (Calculus)	63	40.4%
Not collected	7	4.5%

(*n*, sample size).

**Table 3:** Dietary intake per day according to sex.

Nutrient	Sex	*n*	Mean	SD	95% CI	*p*-Value
Energy intake (kcal/day)	Boys	75	1683.93	539.46	1561.84–1806.02	0.018 *
	Girls	81	1492.80	454.19	1393.89–1591.71	
Proteins (g)	Boys	75	79.33	23.60	73.99–84.67	<0.001 *
	Girls	81	66.75	21.89	61.99–71.51	
Carbohydrates (g)	Boys	75	186.81	70.15	170.93–202.69	0.043 *
	Girls	81	165.18	62.51	151.57–178.79	
Fiber (g)	Boys	75	13.92	8.03	12.10–15.74	0.794
	Girls	81	13.62	6.20	12.27–14.97	
Lipids (g)	Boys	75	66.69	31.18	59.63–73.75	0.133
	Girls	81	59.87	25.05	54.41–65.34	
Saturated fatty acids (g)	Boys	75	23.34	12.64	20.49–26.21	0.135
	Girls	81	20.61	10.06	18.42–22.80	
Monounsaturated fatty acids (g)	Boys	75	25.81	12.62	22.95–28.67	0.526
	Girls	81	24.58	11.46	22.09–27.08	
Polyunsaturated fatty acids (g)	Boys	75	9.21	5.57	7.94–10.47	0.192
	Girls	81	8.10	4.90	7.04–9.17	
Cholesterol (mg)	Boys	75	281.99	168.24	243.91–320.07	0.028 *
	Girls	81	224.80	154.54	191.14–258.46	
Calcium (mg)	Boys	75	730.28	351.37	650.77–809.81	0.013 *
	Girls	81	599.30	302.49	533.42–665.17	
Phosphorus (mg)	Boys	75	1095.81	331.05	1020.89–1170.73	0.001 *
	Girls	81	928.14	311.90	860.22–996.06	
Iron (mg)	Boys	75	12.30	8.14	10.46–14.15	0.033 *
	Girls	81	9.99	5.05	8.89–11.09	
Potassium (mg)	Boys	75	2126.27	827.68	1938.95–2313.59	0.854
	Girls	81	2102.93	749.16	1939.78–2266.08	
Sodium (mg)	Boys	75	2072.03	1022.17	1840.69–2303.37	0.055
	Girls	81	1757.10	1014.74	1536.10–1978.09	
Zinc (mg)	Boys	75	7.56	2.68	6.95–8.17	0.057
	Girls	81	6.78	2.36	6.27–7.30	
Magnesium (mg)	Boys	75	217.94	91.49	197.24–238.66	0.336
	Girls	81	205.63	66.70	191.10–220.16	
Selenium (µg)	Boys	75	26.51	27.66	20.25–32.77	0.312
	Girls	81	21.99	27.82	15.94–28.05	
Vitamin A (µg RE)	Boys	75	558.19	512.40	442.20–674.20	0.466
	Girls	81	500.06	480.36	395.44–604.67	
Vitamin D (µg)	Boys	75	1.69	2.34	1.16–2.22	0.269
	Girls	81	1.27	2.31	0.77–1.77	
Vitamin E (mg)	Boys	75	9.07	8.82	7.08–11.07	0.188
	Girls	81	7.56	5.14	6.44–8.67	
Thiamine (mg)	Boys	75	1.47	0.89	1.27–1.67	0.016 *
	Girls	81	1.17	0.30	1.03–1.31	
Riboflavin (mg)	Boys	75	1.89	1.01	1.66–2.11	0.008 *
	Girls	81	1.50	0.76	1.34–1.67	
Niacin (mg)	Boys	75	22.72	12.24	19.95–25.50	0.006 *
	Girls	81	18.02	8.61	16.14–19.90	
Pyridoxine (mg)	Boys	75	1.85	1.15	1.59–2.11	0.055
	Girls	81	1.54	0.82	1.37–1.72	
Vitamin B12 (mg)	Boys	75	5.43	9.24	3.34–7.52	0.052
	Girls	81	3.33	2.63	2.76–3.90	
Vitamin C (mg)	Boys	75	80.84	70.01	65.00–96.68	0.552
	Girls	81	87.81	75.35	71.40–104.22	

(*n*, sample size; SD, standard deviation; CI, confidence interval, * variable with significant effect (*p* < 0.05)).

**Table 4:** Dietary intake according to periodontal status.

Nutrient	Periodontal Status	*n*	Mean	SD	*t*	*p*-Value
Energy intake (kcal/day)	Healthy	46	1645.76	452.65	0.977	0.330
	Affected	110	1559.15	524.6		
Proteins (g)	Healthy	46	73.31	18.09	0.174	0.862
	Affected	110	72.59	25.51		
Carbohydrates (g)	Healthy	46	186.89	68.23	1.369	0.173
	Affected	110	170.85	66.16		
Fiber (g)	Healthy	46	15.98	8.26	2.564	0.011 *
	Affected	110	12.84	6.40		
Lipids (g)	Healthy	46	64.55	25.77	0.399	0.691
	Affected	110	62.57	29.35		
Saturated fatty acids (g)	Healthy	46	22.97	11.29	0.740	0.460
	Affected	110	21.48	11.50		
Monounsaturated fatty acids (g)	Healthy	46	26.22	10.89	0.707	0.481
	Affected	110	24.73	12.47		
Polyunsaturated fatty acids (g)	Healthy	46	8.54	4.59	−0.146	0.884
	Affected	110	8.67	5.52		
Cholesterol (mg)	Healthy	46	243.67	171.07	−0.425	0.671
	Affected	110	255.90	160.58		
Calcium (mg)	Healthy	46	675.03	320.09	0.309	0.758
	Affected	110	656.94	338.69		
Phosphorus (mg)	Healthy	46	1041.23	303.52	0.791	0.430
	Affected	110	995.17	342.32		
Iron (mg)	Healthy	46	11.81	5.83	0.844	0.400
	Affected	110	10.80	7.16		
Potassium (mg)	Healthy	46	2239.78	805.00	1.295	0.197
	Affected	110	2061.62	774.74		
Sodium (mg)	Healthy	46	1926.73	1104.25	0.143	0.887
	Affected	110	1900.88	998.45		
Zinc (mg)	Healthy	46	7.53	2.15	1.205	0.230
	Affected	110	7.00	2.68		
Magnesium (mg)	Healthy	46	234.82	98.18	2.397	0.018 *
	Affected	110	201.82	68.55		
Selenium (µg)	Healthy	46	31.41	31.59	2.133	0.035 *
	Affected	110	21.14	25.52		
Vitamin A (µg RE)	Healthy	46	672.94	605.79	2.400	0.018 *
	Affected	110	467.40	429.80		
Vitamin D (µg)	Healthy	46	1.04	1.82	−1.487	0.139
	Affected	110	1.65	2.50		
Vitamin E (mg)	Healthy	46	8.22	5.38	−0.074	0.941
	Affected	110	8.31	7.81		
Thiamine (mg)	Healthy	46	1.39	0.73	0.814	0.417
	Affected	110	1.28	0.79		
Riboflavin (mg)	Healthy	46	1.76	0.81	0.676	0.500
	Affected	110	1.66	0.94		
Niacin (mg)	Healthy	46	21.88	8.92	1.207	0.229
	Affected	110	19.61	11.39		
Pyridoxine (mg)	Healthy	46	1.90	0.87	1.683	0.094
	Affected	110	1.61	1.04		
Vitamin B12 (mg)	Healthy	46	4.02	4.32	−0.381	0.704
	Affected	110	4.47	7.54		
Vitamin C (mg)	Healthy	46	107.08	86.65	2.558	0.011 *
	Affected	110	75	64.08		

(*n*, sample size; SD, standard deviation; *t*, *t*-value, Student’s *t*-test; * variable with significant effect (*p* < 0.05)).

**Table 5:** Dietary Reference Values (EFSA) [22,23,24,25,26,27].

	Fiber	Magnesium	Selenium	Vitamin A	Vitamin C
Boys	21 g	300 mg	70 µg	750 μg RE	100 mg
Girls	21 g	250 mg	70 µg	650 μg RE	90 mg

**Table 6:** Nutrient intake and percentage of EFSA Dietary Reference Values met, stratified by periodontal status and sex.

Periodontal Status	Sex	*n*	Fiber (g; %DRV)	Magnesium (mg; %DRV)	Selenium (µg; %DRV)	Vitamin A (µg RE; %DRV)	Vitamin C (mg; %DRV)
Healthy	Boys	21	16.2 ± 2.4 (77.0%)	239.3 ± 28.8 (79.8%)	33.6 ± 6.6 (48.0%)	760.0 ± 169.3 (101.3%)	77.2 ± 13.0 (77.2%)
	Girls	25	15.8 ± 1.1 (75.3%)	231.1 ± 11.9 (92.4%)	29.6 ± 6.6 (42.3%)	599.8 ± 83.5 (92.3%)	132.2 ± 19.7 (146.9%)
Periodontal disease	Boys	54	13.0 ± 0.9 (62.1%)	209.7 ± 9.5 (69.9%)	23.8 ± 3.6 (33.9%)	479.7 ± 46.6 (64.0%)	82.3 ± 10.1 (82.3%)
	Girls	56	12.6 ± 0.8 (60.2%)	194.3 ± 9.0 (77.7%)	18.6 ± 3.3 (26.6%)	455.6 ± 67.2 (70.1%)	68.0 ± 7.0 (75.5%)

Intake data are presented as mean ± SE. Abbreviations: DRV, Dietary Reference Value; EFSA, European Food Safety Authority; RE, retinol equivalents.

**Table 7:** Proportion of adolescents meeting the EFSA Dietary Reference Values, according to periodontal status.

Nutrient	Healthy (*n* = 46)	Affected (*n* = 110)
Fiber	8 (17.4%)	15 (13.6%)
Magnesium	12 (26.1%)	13 (11.8%)
Selenium	5 (10.9%)	9 (8.2%)
Vitamin A	17 (37.0%)	16 (14.5%)
Vitamin C	22 (47.8%)	31 (28.2%)

**Table 8:** Nutrient deficits relative to the EFSA Dietary Reference Values according to periodontal status.

Nutrient	Healthy (Mean ± SD)	Affected (Mean ± SD)	*p*-Value
Fiber (g)	−5.02 ± 8.26	−8.16 ± 6.40	0.011 *
Magnesium (mg)	−38.01 ± 100.33	−72.72 ± 70.30	0.015 *
Selenium (µg)	−38.59 ± 31.59	−48.86 ± 25.52	0.035 *
Vitamin A (µg RE)	−22.72 ± 601.16	−258.23 ± 334.89	0.002 *
Vitamin C (mg)	+12.51 ± 88.39	−19.91 ± 63.71	0.011 *

Data are expressed as mean ± standard deviation (SD). Comparisons between groups were performed using Student’s *t*-test for independent samples. Negative values indicate intake below the EFSA Dietary Reference Values, whereas positive values indicate intake above them. * *p* < 0.05.

**Table 9:** Binary logistic regression analysis of factors associated with periodontal disease.

Variable	OR (95% CI)	*p*-Value
Sex	0.875 (0.416–1.843)	0.726
Energy intake	1.001 (1.000–1.002)	0.232
Dietary fiber	0.994 (0.917–1.077)	0.885
Magnesium	0.997 (0.990–1.004)	0.457
Selenium	0.987 (0.973–1.000)	0.056
Vitamin A	0.999 (0.998–1.000)	0.096
Vitamin C	0.995 (0.989–1.000)	0.068

## Data Availability

The datasets generated and/or analyzed during the current study are not publicly available as they are being utilized for ongoing purposes, but they are available from the corresponding author upon reasonable request.

## Abbreviations

The following abbreviations are used in this manuscript:

CAL	Clinical Attachment Loss
CEI-IB	Research Ethics Committee of the Balearic Islands
CI	Confidence Interval
CPI	Community Periodontal Index
DRV	Dietary Reference Value
EFSA	European Food Safety Authority
EU Menu	European Union Menu Project
Mg/Ca	Magnesium-to-Calcium Ratio
OR	Odds Ratio
PPD	Periodontal Probing Depth
SD	Standard Deviation
WHO	World Health Organization
